# Keto microbiota: A powerful contributor to host disease recovery

**DOI:** 10.1007/s11154-019-09518-8

**Published:** 2019-11-13

**Authors:** Amanda Cabrera-Mulero, Alberto Tinahones, Borja Bandera, Isabel Moreno-Indias, Manuel Macías-González, Francisco J. Tinahones

**Affiliations:** 1 Deparment of Endocrinology and Nutrition, Virgen de la Victoria University Hospital, Institute of Biomedical Research in Malaga (IBIMA) and University of Malaga, Campus de Teatinos s/n, 29010 Malaga, Spain; 2 CIBER Physiopathology of Obesity and Nutrition (CIBERobn), Institute of Health Carlos III, Madrid, Spain

**Keywords:** Gut microbiota, Dysbiosis, Ketosis, Ketogenic diets, Keto microbiota, Metabolites

## Abstract

Gut microbiota (GM) is a key contributor to host metabolism and physiology. Data generated on comparing diseased and healthy subjects have reported changes in the GM profile between both health states, suggesting certain bacterial composition could be involved in pathogenesis. Moreover, studies reported that reshaping of GM could contribute actively to disease recovery. Interestingly, ketogenic diets (KD) have emerged recently as new economic dietotherapeutic strategy to combat a myriad of diseases (refractory epilepsy, obesity, cancer, neurodegenerative diseases…). KD, understood in a broad sense, refers to whatever dietetic approximation, which causes physiological ketosis. Therefore, high fat-low carbs diets, fasting periods or caloric restriction constitute different strategies to produce an increase of main ketones bodies, acetoacetate and β-hydroxybutyrate, in blood. Involved biological mechanisms in ketotherapeutic effects are still to be unravelled. However, it has been pointed out that GM remodelling by KD, from now on “keto microbiota”, may play a crucial role in patient response to KD treatment. In fact, germ-free animals were resistant to ketotherapeutic effects; reinforcing keto microbiota may be a powerful contributor to host disease recovery. In this review, we will comment the influence of gut microbiota on host, as well as, therapeutic potential of ketogenic diets and keto microbiota to restore health status. Current progress and limitations will be argued too. In spite of few studies have defined applicability and mechanisms of KD, in the light of results, keto microbiota might be a new useful therapeutic agent.

## Gut microbiota: A prokaryotic organ with multiple functions in the human body

Gut microbiota constitute a complex and dynamic ecosystem formed by bacteria, archaea, viruses, and fungi [1]. However, bacteria are the most studied [1]. The gastrointestinal tract is one of the widest surface area in the human body [2], and probably, the most diverse microbiota within the human body [3]. Microbiota members interact between them and with the host on multiple levels.

It is known microbiota and their metabolites are able to influence our physiology, both in health and disease [3]. Microbiota participates in host digestion and nutrition by producing nutrients from non-digestible substrates, contributes to metabolic functions, protects against pathogens, modulates the immune system, synthetizes vitamins, and produces a wide variety of biochemically active compounds (including neurotransmitters and enzymatic cofactors) [2, 3], among others. Therefore, through these vital functions, gut microbiota influences our physiology.

Multiple metagenomic data generated on comparing feces from diseased and healthy subjects have reported changes in the gut microbiota profile between both health states. Increasing observations have described a significant correlation between an imbalance of the microbiota profile, or dysbiosis, and the development of certain diseases [1, 4]. In fact, gut microbiota changes have been defined during obesity, diabetes, liver diseases, cardiometabolic disorders, inflammatory bowel diseases, autoimmune conditions, cancer [1, 2], and even nervous system disorders (autism, anxiety, depression, multiple sclerosis, Parkinson’s disease, and Alzheimer’s disease) [5].

**Fig. 1:**
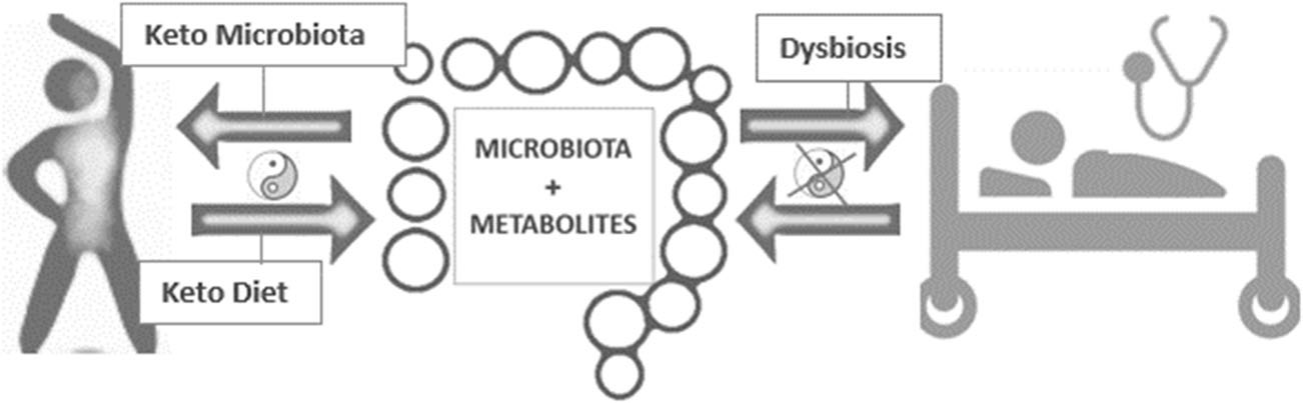
Gut microbiota is a key contributor to host metabolism and physiology. Ketogenic diet and/or keto microbiota might restore health state by regulating multiple mechanisms, which remain unknown*. Yin Yang* symbol represents host-microbial mutualism

The altered microbiota profile in different diseases, as well as its putative implication in the physiopathology of these diseases, has positioned to the gut microbiota as a potential source of innovative therapeutics [1]. However, the symbiotic relationship between gut microbiota and its host has triggered in different profiles among individuals [3]. As result, there is not a consensus about the optimal microbiota profile, which has the potential of protecting against any disease. However, many efforts have been put for this purpose Fig. 1.

## Modulation of the gut microbiota profile

Gut microbiota composition is influenced by several factors, both genetic and environmental: human genetic, mode of delivery, type of feeding, medication (laxatives, antibiotics, proton pump inhibitors, metformin [6]), stress, infections, smoking, physical inactivity, bariatric surgery, fasting … [7–9].

However, diet is one of the main drivers of microbiota changes [6]. The overall features of the diet (i.e., total calories, timing, variety of nutrients, vitamins and fiber ingestion, percentage of macronutrients,…) all influence the composition of the microbiota and can modify it in short time [6]. Interestingly, chrononutrition has become extremely important as modulator of microbiota, since disruption of circadian rhythms can increase the risk of disease [10]. It is known that microbiome is affected by what is eaten but also by when food is consumed [11]. Time of eating has been reported to restore circadian rhythms affecting bacterial communities and its function [10–12]. In fact, time-restricted feeding (TRF), an eating pattern in which food intake is restricted to a time window which can oscillates between 8 and 12 h [13, 14], is able to restore the cycling of the microbiota [11]. Therefore, restoring circadian rhythms, including microbiota rhythm, may also contribute to optimize individual’s physiology and decrease their risk of disease [10].

Undoubtedly, microbes that reside in the human gut are key contributors to host metabolism and, consequently, they are considered potential therapeutic targets [1]. For this reason, prebiotics, probiotics and fecal microbiota transplantation have emerged as new opportunities to promote and maintain a healthy microbiota and consequently a healthy homeostasis:

- **Probiotics** term refers to *“administration of live microorganisms in adequate amounts able to confer a health benefit on the host”.* Consumption of live microbiota in both foods and dietary supplements has been related to amelioration or prevention of intestine inflammation and other intestinal or systemic disease phenotypes [15–18]**.**
- **Prebiotics** are non-digestible selectively fermented dietary fibres that specifically promote the growth of beneficial targeted bacteria in gut providing some kind of benefit to the host, like immune regulation [19].
- **Fecal microbiota transplantation (FMT)** has been reported as a useful medical tool, for example, in treating *Clostridium difficile* infection or insulin-resistance in obese patients [2], but, it is necessary yet to optimize the process and make donor microbiota perdurable in receptor.

However, despite the fact **diet** is the strongest and safer influencer on gut bacterial communities, few clinical studies of different kind of dietary interventions on human gut microbiota have been reported so far [2, 20]. Therefore, in this review we discuss the effect of different diet interventions on gut microbiota, focusing on a current science hot-topic, *“the ketogenic diet”.*

## Keto diet and potential benefits of *“keto microbiota”*

Although ketogenic diets debuted in 1920 as a medical strategy to treat refractory epilepsy, its healing properties had not been scientifically evaluated in other medical areas [21, 22]. Recently, its applicability and efficacy are being tested in the treatment of other diseases such as obesity [23], polycystic ovarian syndrome [23], cancer [24], cardiovascular problems [25] and respiratory problems [26]. Multiple worldwide open clinical trials evaluate the tolerability and efficacy of ketogenic diets in the treatment of the aforementioned pathologies as a new medical treatment (http://ClinicalTrials.gov).

KD, understood in a broad sense, refers to any dietetic approximation able to produce a physiological ketosis, this is, an increase of ketone bodies. Classic ketogenic diets [21], fasting periods [27], time restricted feeding [13], caloric restriction diets [28], or intense physical exercise [29] constitute different strategies to produce ketonemia (increase of main ketones bodies, acetoacetate and β-hydroxybutyrate, in blood). Moreover, supplements that mimic the ketosis state as ketone esters [30] or ketone salts [31] have been developed in an attempt to overcome disadvantages of KD without a modification of the diet.

However, little is known about the underlying mechanisms of action of KD. The most accepted hypotheses point out: metabolic changes, alteration of the signalling pathways, changes in the production of hormones and neurotransmitters, epigenetic modifications [22, 24, 27], and as would be expected, modulation of the microbiota [32].

As we mentioned above, the gut microbiota plays an intermediary role between diet and host physiology. Diet affects composition, diversity and functionality of the gut microbiota and these changes in the gut microbiota are inducible and reproducible [33].

Currently, few data is available about the effects of ketogenic diets on gut microbiota composition [34]. *(In this review, we have used the term “keto microbiota” to define a profile of gut microbiota moulded by a keto diet).* Most of existing data about the KD impact into microbiota comes from epilepsy studies. Recent studies have reported that gut microbiota changes induced by a KD are required to improve the symptomatology of some diseases such as autism [35], epilepsy [33], or sclerosis [36].

### Ketogenic diet, keto microbiota and epilepsy

The classic ketogenic diet (CKD) is a high-fat, adequate-protein, low-carbohydrate diet [21]. The most common ratio in this diet is 3:1 or 4:1. That is, 80–90% of the energy comes from fat and 10–20% from the combination of carbohydrates and proteins [24]. The term was coined by Wilder in 1921 who found that fasting caused an improvement in their epileptic patients and tried to mimetic the ketosis state provoked by fasting with a very low carb diet [37]. Since then, CKD has been the treatment of choice in epileptic refractory patients [21].

Gut microbiota profile is significantly different between healthy and epileptic individuals. KD treatment is able to reshape gut microbiota in humans and rodents [38, 39]; and this keto microbiota is required to avoid seizures. In fact, mouse models of refractory epilepsy showed that those given antibiotics or reared in a germ-free environment were resistant to seizure protection from KD, while keto microbiota fecal transplant helped mice with seizure control. Therefore, these results support that keto microbiota is necessary to protect against seizures [32, 40].

Interestingly, after a KD intervention, patients were differentiated into responder or non-responder subjects according to their gut microbiota changes, suggesting that the effectiveness of a KD was driven by the gut microbiota [5, 32]. Moreover, responder and non- responder groups differed in gut bacteria profiles at the level of order, family and genus, but also in microbial metabolites production. Such bacterial metabolites could be act by restricting precursors availability to synthetize inhibitory neurotransmitters involved in seizure control [40].

In parallel, Hampton et al. revealed that certain combinations of bacteria are required to improve epileptic symptomatology, for instance: they exposed that *“Akkermansia muciniphila and Parabacteroides sp. colonization together but not alone protected against seizures in germ-free mice fed the ketogenic diet”* [40]. Taken together, these findings underlie that microbiota is a complex system, where interactions between different species enable generate determined profiles of metabolites responsible to provoke a physiological response in host.

By contrast, in spite of benefits of keto microbiota in a growing number of diseases, Tagliabue reported that prolonging the KD for 3 months could cause dysmicrobism with damage to the gut health [41]. Consequently, they recommended prebiotics or probiotics treatment to re-establish gut microbiota and intestine homeostasis [41]. However, more follow-up studies are required in order to monitor the changes of the microbiota profiles with KD, and this highlights the necessity to monitor side effects and take into account possible dysbiosis.

## Different ketogenic diets lead to a different gut microbiota

Although more studies are required to compare microbiota profile between different KD, in the light of outcome, probably, both microbiota profile and physiological responses may be dissimilar. Currently, results regarding microbial communities profile and diversity are controversial.

High fat, adequate protein, low carbs diet (CKD) seems to be associated with a lower diversity; finding which could be justified because microbiota is responsible to degrade undigested carbohydrates [2, 3], which are diminished in this type of diet.

By contrast, intermittent or continuous reduction of the caloric intake, caloric restriction (CR), might not affect *alpha*-diversity [42, 43]. In spite of this, CR has been reported to produce significant changes in faecal bacteria composition and metabolite content [4, 43], thus, affecting gene expression related to metabolism and inflammation [43].

For its part, fasting periods affect clock gene transcription, [44] as well as, gut microbiota itself [45]. Disturbance of the intestinal circadian clock lead to change the uptake of nutrients, gut motility, hormones production, gut microbiota fluctuations, and ultimately, the whole body and its microbiome [43]–[45]**.**

Main changes produced in the gut microbiota by KD are reflected in the Table 1 (below shown).

**Table 1:** main gut microbiota changes produced by KD treatment. Generally, CKD is related to diversity decrease [5, 33, 36], while, CR did not decrease diversity [42, 43]. CKD: Classical ketogenic diet (high fat, adecuate protein low carbs). CR: caloric restriction or energy restriction

Type of KD	Human vs animal	Disease	KD duration	Phylum	Family	Genus	Species	Ref
CKD	Human (children)	Refractory epilepsy Responders	6 months	Increase *Bacteroidetes.* Decrease *Firmicutes* and *Actinobacteria*				[5]
CKD Zeneca	Human (children)	Refractory epilepsy Responder	1 week	Increase *Bacteroidetes.* Decrease *Proteobacteria*				[39]
CKD	Human	GLUT1 deficiency syndrome	3 months				Increase *Desulfovibrio spp*	[41]
CKD	Mouse	Refractory epilepsy	14 days		Increase *Erysipelotrichaceae*	Increase *Akkermansia ParabacteroidesSutterella*	Increase *Akkermansia muciniphila*	[33]
CKD	Human	Multiple Sclerosis	6 months			Increase *Akkermansia*		[36]
CKD	Mouse	Autism	14 days	Increase *Bacteroidetes*			Decrease *Akkermansia muciniphila*	[35]
CKD	Mouse	Refractory-epilepsy	14 days		Increase *Erysipelotrichaceae*	Increase *Parabacteroide, Sutterella* *Decrease Allobaculum, Bifidobacterium,* and *Desulfovibrio*	Increase *Akkermansia muciniphila,*	[40]
CR VLCD	Human	obesity	4 weeks	Decrease *Proteobacteria*		Increase *Ruminococcus* and *Bifidobacterium*	Increase *Anaerostipes hadrus* Decrease *Agathobacter rectalis*	[42]
CR 25%	Mouse		14 days	Decrease *Actinobacteria* and *Proteobacteria*	Increase *Lactobacillaceae, Lachnospiraceae, Ruminococcaceae and Erysipelotrichaceae*	Decrease *Clostridiales*		[43]
Fasting periods	Human		1 week				Increase *Lactobacilli, Enterobacteria,* and *Akkermansia.*	[46]
Fasting	Hamster		96 h	Increase *Proteobaacteria Verrucomicrobia*	Increase *Desulfovibrionaceae*		Increase *Akkermansia*	[47]

Since Zhang et al. reported different gut microbiota profile after KD between responder and non- responder epileptic patients [5], it is necessary to understand how there are different microbiota profiles more susceptible to be changed by KD than others. In addition, we must comprehend how a KD can overcome this challenge to prescribe it as a medical treatment.

## Benefitial effects of keto diet: Is microbiota the only responsible for?

Previously, some studies reported that microbiota by itself was sufficient to enhance insulin sensitivity, improve tolerance to glucose and cold, and reduce fat content [48].

However, surely ketotherapeutic effects are result from different mechanisms: microbiota, epigenetic, metabolic reprogramming… Those all components could act interdependent and interrelated to many others.

Importantly, Ketone bodies (KB) fulfil several functions: 1) KB are energetic substrates, which are oxidized in heart, brain, and muscle during ketotic states, therefore, participating in bioenergetic homeostasis; 2) KB are also anabolic substrates contributing to lipogenesis and sterol biosynthesis in developing brain and lactating mammary gland, among other tissues;3) KB control mitochondrial metabolism and energetics; 4) KB reduce oxidative stress by inhibiting ROS/superoxide production, preventing lipid peroxidation and protein oxidation and increasing antioxidant protein levels; 5) KB act as signalling intermediators; 6) KB modulate inflammation and immune cell function; 7) KB decrease cellular damage, injury, death and lower apoptosis in neurons, lung cells and cardiomyocytes; 8) KB activate a reprogramming of pancreatic islet cells and could be involved in improved β-cell outcomes in states of increased ketogenesis, [49–53], and so on. Likewise, microbiota is involved in the regulation of multiple host metabolic pathways, giving rise to interactive host-microbiota metabolic, signalling, and immune-inflammatory axes that physiologically connect the gut, liver, muscle, heart, kidney and brain [54]. Therefore KD exert a double effect about microbiota, directly by modifying substrate availability and indirectly modifying several functions which also impacts on microbiota.

In the same line, epigenetic changes can be influenced by microbiota, but also by ketone bodies. Ketone bodies (β-hydroxybutyrate and acetate) have been confirmed to affect epigenetic mark by inhibiting histone deacetylase class I [55], modifying proteins at the post-translational level by butyrylation [56], affecting DNA methylation [57] and acetylating histone and non-histone proteins [58]. But, metabolites produced by microbiota are also substrates or cofactors of enzymes involved in epigenetic process [59]. Therefore, it is not possible to discern epigenetic changes derived from both contributors.

Likewise, microbiota is a known endocrine organ which produces hormones and bioproducts with effects on the hosts’ biology [60]. But, microbiota composition is also influenced by hormones such as estrogens [61], so, there is a mutual crosstalk. KD have shown to affect both, microbiota and several hormones levels [13, 62]. Although to date, results about importance of gender on ketotherapeutic effects are controversial [63–65], due to the interplay between diet-microbiota-hormones, the sex and hormonal status of an individual may influence on the efficacy of keto treatments. Reinforcing this, KD has become as a promising treatment in those diseases where sex hormones profile is altered such as ovarian or endometrial cancer [51], obesity [65] or polycystic ovarian syndrome [51].

**Fig. 2:**
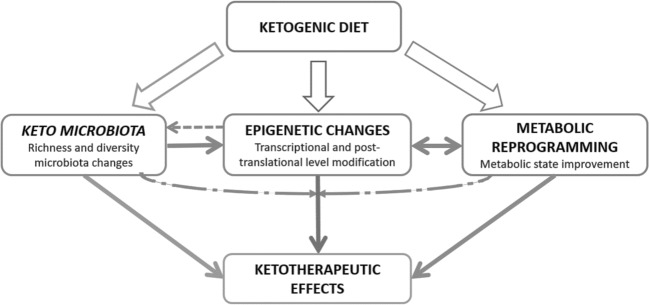
Ketogenic diets may modulate microbiota profile, epigenetic mark, and metabolic reprogramming, as well as many others. Those components could act interdependent and interrelated to many others. Exact biological mechanisms responsible for ketotherapeutic effects are still to be unravelled

Similarly, metabolic reprogramming may be the result of changing the microbiota profile [66], but also as a consequence of forcing to the organism to use ketone bodies like energy substrate [67]. Inflammation decrease may be result of lower content of pro-inflammatory bacteria [1], but also, consequence of lower infiltration of macrophages in adipose tissue due to fat loss derived of energy restriction [68]… so, this linking scenario could be observed with each of hypothesized mechanisms Fig. 2.

Below is a Table 2 summarising reported effects of ketotherapeutic therapies, as well as, putative involved biological mechanisms. Casual relationships are not defined yet. Additional genomics, metabolomics, and proteomics studies are required to identify molecular mechanisms through which ketosis regulates physiology responses and nuclear signalling events in each disease.

**Table 2:** Beneficial therapeutic effects of KD in different diseases and defined biological mechanisms, which could be involved in advantages of KD

Type of KD tested	Disease	Effect	Involved biological mechanisms allegedly derived by KD
Fasting Classic ketogenic diet	Refractory epilepsy	Seizure protection [22, 39, 68]	Increase gamma-aminobutyric acid (GABA) Decrease glutamate pH alteration affecting excitability GABA Microbiota profile reshape
Caloric restriction Classic ketogenic diet Fasting periods	Obesity	Weight loss and metabolic profile improvement [48, 68–73]	Appetite suppression by ketone bodies Gut microbiota profile and its metabolites change *Beiging* of white adipose tissue Brown adipose tissue activation (increase uncoupling protein UCP1 expression) Loss of fat (not lean mass) Increase metabolic cost of gluconeogenesis Increase lipogenesis and decrease lipolysis Metabolic improvements, browning and fat loss associated with microbiota remodelling
Caloric restriction Classic ketogenic diet	DT2	Improve insulin sensitivity and glycaemic control [68, 74, 75]	Decrease inflammation Reduce insulin Lipid profile improvement Decrease body weight, glycaemia, glycosylated haemoglobin, triglyceride level and LDL cholesterol Increased HDL cholesterol levels Greater weight loss than other diets in the short term Remarkable metabolic improvement Reduced or even withdrawn antidiabetic treatment
Classic ketogenic diet Ketone supplements	Alzheimer	Improve motor performance and cognition [30, 76–78]	Compensate for the deficiency in glucose metabolism Decrease glutamate Increase GABA Decrease reactive oxygen species (ROS) Decrease Inflammation Enhancing metabolism Metabolic state improvement Increase neurovascular integrity Application of theory brain-gut-microbiota axis
Classic ketogenic diet	Multiple Sclerosis	Normalized concentrations of the colonic microbiome [36]	Restore colonic microbiome Application of theory brain-gut-microbiota axis
Classic ketogenic diet	Autism	Mitigate some of the neurological symptoms associated with Autism Spectrum Disorder [35, 79]	Application of theory brain-gut-microbiota axis
Fasting	Cardiovascular	Improve cardiovascular health [80]	Supply of ketone bodies to the heart where KBs are efficiently oxidized Synergic actions between butyrate production by gut bacteria and circulating high blood ketones Application of theory gut–heart metabolic axis
Caloric Restriction Classic ketogenic diet	Cancer	Tumor growth decrease Increase survival patient [67, 81–83]	Cancer cell starvation (glucose privation reduce energy production of cancer cells) Repress Warburg effect Decrease inflammation Decrease insulin and insulin growing factor Decrease angiogenesis Decrease PI3K/Akt signalling (a known pathway involved in carcinogenesis) Increase apoptosis Affect tumor epigenetic Avoid cachexia (inhibition of muscle and body weight loss) Sensitize cancer cells to oncological treatment

## Putative therapeutic scope of ketogenic diets: Keto microbiota is sought

Ketogenic diets are being tested in degenerative and non-neurodegenerative diseases with successful results, although gut microbiota profile was not assessed. Here, we make one little sweep about last therapeutic findings of KD where it would be interesting to study keto microbiota.

### Ketogenic diet and cancer

KD has been linked with a decrease in tumour growth [81] and an increase of the patient survival [84]. Currently, KD has been particularly suitable for adjuvant tumour therapy able to sensitize tumor cells to conventional chemotherapeutic and radiotherapeutic treatments [24, 85]. Recent studies also showed dietary administration with keto-formula could suppress tumor progression, improve systemic inmune responses and body composition, which might help to prevent cancer cachexia [82].

Probably, keto microbiota could play an important role in some kinds of cancer due to microbiome is able to establish connecting axes with other organs.

In fact, microbiota composition differs between healthy and cancer subjects, whereby, certain microbial genes can be used as cancer diagnostic biomarkers [86]. Murine experiments confirmed that germ-free mice had significant lower risk of developing colon tumours, suggesting microbiota could participate in first steps of carcinogenesis [87].

Reinforcing this, Klemment et al. underlined that there is a complex network between cancer, diet and microbiota [88]. Dietary habits have been associated with a remarkable figure of cancers [86]. The kind of consumed diet could tip the balance towards a type of microbiota able to produce oncometabolites or tumor-suppressive metabolites [89]. Both, microbiota and KD are responsible to produce short-chain fatty acids (SCFAs), whose role on cancer prevention and treatment have been broadly demonstrated. Therefore, KD and keto microbiota could contribute synergistically to prevent tumorigenesis scenario [88, 89]. Moreover, microbiota could also affect immunotherapy response and toxicity in oncological patients since microbiota modulate host immunity [90].

As a result, KD dietary treatment and/or keto microbiota could constitute promising strategies to curb carcinogenesis and to increase effectiveness of oncological therapies.

### Ketogenic diet and neurodegenerative diseases

The beneficial effects of ketogenic diets have been shown in a wide variety of these neurological disorders such as Parkinson’s, Alzheimer’s and traumatic brain damage [71, 76]. In short, any neurodegenerative disorder characterized by neuronal hypometabolism can be approached with ketosis-producer diets since ketone bodies provide an alternative source of energy for hypometabolic neurons [91]. Similarly, it has been pointed out that KD could contribute to the overcoming of the cognitive deficits that occur with aging through an increase in capillary density and levels of hypoxia inducible factors [92]. Considering, gut microbiota and neuropathology are closely interrelated [36], surely defining microbiota profile will help to define a subset of neuropathologies which can be afforded by keto diet or keto microbiota.

Several studies have announced gut dysbiosis is related to neurological disease [78, 93]. But, at the same time, gut microbiota composition can be also affected by some mental disorders [93].

At present, It is well defined the bidirectional link between neurological diseases and gut microbiota alterations [78], reinforcing the the gut–brain axis theory. Microbiota could contribute to maintain neural homeostasis by different mechanisms such as: 1) producing active biological compounds, 2) interacting with intestinal barrier, 3) connecting neuro-endocrine-immune system [78], or 4) direct neuronal communication [93]. The disruption of one of these mechanisms could increase inflammation which constitutes one of the main contributors to neurodegeneration status [93].

Therefore, it is fair to say keto microbiota could play a crucial role in the ketotherapeutic responses.

(Note: Diseases mentioned in Table 2 also require analysing gut microbiota and its metabolites to shed more light about pathways and networks, which are stablished between keto microbiota and other factors).

## Some considerations and limitations about available literature

- Keto microbiota resilience. So far, there is a broad lack of knowledge about remodelling capacity of microbiota after stopping KD treatment. Likewise, most studies have focused on gut microbiota. However, microbiota in other body sites could also modify by keto diet and could participate in disease recovery, so more studies are required in this field. Studying these events would contribute to improve biotherapies, even, its medical prescription to treat certain pathologies.
- Keto microbiota study follow-up. Most studies have characterized gut microbiota prior and after KD treatment. However, considering proper treatment duration is not established yet, maybe this final point of study is not representative of the most therapeutic capacity of KD. In this sense, monitor gut microbiota periodicity could contribute to understand benefits of ketotherapy.
- Keto microbiota analysis corrected by other confusing variables: On the other hand, classic ketogenic diet (high lipids, low carbohydrates, adequate proteins) has been reported to lead to digestive problems due to the high fat content and deficiencies of minerals, vitamins and electrolytes by restringing the consumption of fruits and vegetables [94]. For this reason, vitamin and mineral supplements ingestion are recommended to alleviate symptoms and signs associated with the deficiencies of these compounds [95, 96]. Therefore, we wonder whether studies about CKD treatment were supported with vitamins, because microbiota profile could vary based on this. Thus, microbiota changes described could not be just the result of ketosis state. Moreover, microbiota profile should be also corrected by other confusing factors (smoking, alcohol, exercise…)
- Do KD affect keto microbiota diversity? So far, few human studies have analysed changes in microbiota and its metabolites induced by KD and data are controversial. There are how confirm KD treatment reduce diversity, while, others affirm that there are not changes in richness and variety microbial communities. Probably, these inconsistencies are result of non-standardized protocols and differences in KD treatment duration, age of study population, base disease, or type of KD, among others. Surely, gut microbiota changes induced by caloric restriction could be different from those that are produced by a high fat-low carbs diet. Since, different dietetic approximations influence on a diverse way, as we commented above. Therefore, the type of diet could modulate a keto microbiota, more than ketosis state per se*.*
- Effects of ketone supplements on microbiota: It would be interesting to test whether ketone supplements can modulate microbiota profile. This approach would represent a way to understand whether ketotherapeutic effects are just the result of ketosis or whether an additional microbial pattern is required to get these health benefits.

## Concluding remarks

- Keto diet is the treatment of choice in refractory epilepsy but KD also seems to be a diet-therapeutic strategy with vigorous potential in a myriad of diseases such as cancer, metabolic and endocrine diseases and neurological disorders.
- Gut microbiota is a key contributor to host metabolism and physiology. Gut microbiota profile varies among healthy and sick individuals, and certain bacterial communities have been associated with higher risk of disease.
- KD can reshape gut microbiota profile, and this microbiota modulation is able to generate a remarkable improvement in some diseases such as epilepsy or autism.
- Ketogenic diets modulate microbiota profile, epigenetic mark, and metabolic reprogramming, as well as many other mechanisms. Those components could act interdependent and interrelated to many others.
- Further studies will confirm whether microbiota profile and ketophysiological responses varies between different ketotherapeutic strategies. Currently, results regarding keto microbial communities profile and diversity are controversial.
- Ketogenic diets together with chrononutrition may play a key role in restoring circadian rhythms and microbiota cycling, protecting from potential diseases or improving disease outcome.
- Established axis between diet-microbiota and different tissues highlights the synergic activation of different mechanisms to restore health status, reinforcing the crucial and intermediate role of microbiota for this.

In conclusion, ketogenic diets seem to be a promising new therapy, although, it is early to know its effectiveness and scope. Considering diet is the main modulator of gut microbiota, then, keto microbiota could be a key factor involved in ketotherapeutic effects since microbiome works a vital network between different organs. Nevertheless, more clinical and preclinical studies are required to define the ideal KDs exposure time, or failing that, “keto microbiota profile”, to achieve proper functioning of the body in different pathologies.
